# Towards designing reactive glasses for alkali activation: Understanding the origins of alkaline reactivity of Na-Mg aluminosilicate glasses

**DOI:** 10.1371/journal.pone.0244621

**Published:** 2020-12-30

**Authors:** Harisankar Sreenivasan, Wei Cao, Yongfeng Hu, Qunfeng Xiao, Mohsen Shakouri, Marko Huttula, John L. Provis, Mirja Illikainen, Paivo Kinnunen

**Affiliations:** 1 Faculty of Technology, Fibre and Particle Engineering Research Unit, University of Oulu, Oulu, Finland; 2 Faculty of Science, Nano and Molecular Systems Research Unit, University of Oulu, Oulu, Finland; 3 Canadian Light Source Inc., Saskatoon, Canada; 4 Department of Materials Science and Engineering, The University of Sheffield, Sheffield, United Kingdom; Chiang Mai University, THAILAND

## Abstract

Alkali-activated materials (AAMs), sometimes called geopolymers, are eco-friendly cementitious materials with reduced carbon emissions when compared to ordinary Portland cement. However, the availability of most precursors used for AAM production may decline in the future because of changes in industrial sectors. Thus, new precursors must be developed. Recently there has been increased interest in synthetic glass precursors. One major concern with using synthetic glasses is ensuring that they react sufficiently under alkaline conditions. Reactivity is a necessary, although not sufficient, requirement for a suitable precursor for AAMs. This work involves the synthesis, characterization, and estimation of alkaline reactivity of Na-Mg aluminosilicate glasses. Structural characterization showed that replacing Na with Mg led to more depolymerization. Alkaline reactivity studies indicated that, as Mg replaced Na, reactivity of glasses increased at first, reached an optimal value, and then declined. This trend in reactivity could not be explained by the conventional parameters used for estimating glass reactivity: the non-bridging oxygen fraction (which predicts similar reactivity for all glasses) and optical basicity (which predicts a decrease in reactivity with an increase in Mg replacement). The reactivity of the studied glasses was found to depend on two main factors: depolymerization (as indicated by structural characterization) and optical basicity. Depolymerization dominated initially, which led to an increase in reactivity, while the effect of optical basicity dominated later, leading to a decrease in reactivity. Hence, while designing reactive synthetic glasses for alkali activation, structural study of glasses should be given due consideration in addition to the conventional factors.

## 1. Introduction

The Portland cement (PC) industry is considered a critical part of human civilization in the sense that it is used to produce concrete, which is the resource that humans use most after water, in terms of total volume consumed annually [1]. Cement production has been significantly rising over the past few decades, and it is expected that production volume will double by 2050, mostly because of increased demand in developing economies [2]. However, it is also a fact that the cement industry is responsible for roughly 6%-8% of the global CO_2_ emissions [3,4]. Nearly half of these emissions are related to raw materials; they arise because of the calcination of CaCO_3_. The other half originates from fuel combustion and transport [5,6]. Considering the significance of the CO_2_ emissions from the cement industry sector, there is a growing focus on the development of alternative cementitious materials with reduced carbon footprints.

Alkali-activated materials (AAMs), including those named as geopolymers, are projected to be eco-friendly cementitious materials with reduced carbon footprints [7–9]. They can be tailored to provide a wide array of properties such as high compressive strength, low shrinkage, fast or slow setting, acid resistance, fire resistance, and low thermal conductivity [10–13]. Because of this, they can substitute for PC in applications related to civil infrastructure, and they might be used in niche applications including the production of lightweight materials, as well as cements for underground use, high temperature applications, and as a stabilization/solidification matrix for hazardous or radioactive wastes [7]. AAMs are prepared by alkaline activation of aluminosilicate precursors, which mostly include fly ash, blast furnace slag (BFS), and metakaolin. As fly ash and BFS are by-products from other industries, they do not contribute to the CO_2_ emission related to raw materials, unlike the CaCO_3_ used to manufacture PC. However, there are some disadvantages associated with these precursors. Most of them are fully used; they are available only in certain regions, and their available volumes may decline further because of changes in industrial practices [2,14–16]. Hence, to ensure a sufficient supply of reactive aluminosilicates for AAM production, new materials must be developed.

Recently, interest has grown in the development of synthetic glasses as supplementary cementitious materials [15,17–21]. Compared to PC, synthetic glasses can be produced with slightly lower fuel-related CO_2_ emissions, but significantly lower raw material-related CO_2_ emissions [22]. The obvious advantage of using synthetic glasses for AAM production is that unlike waste-derived raw materials, their composition and properties can be easily tuned. This allows for tailoring the final AAM properties for the specific application under consideration. Most of the synthetic glasses studied contained significant quantities of Ca derived from CaCO_3_, which contributes to carbon emissions. One possible way to further reduce carbon emissions is to replace Ca with Mg. Mg could be derived from non-carbonate precursors like olivine or talc, and those precursors can also serve as sources of silicon. In that way, aluminosilicate glasses that contain Mg are attractive in the context of alkali activation. The rationale for choosing Na as an additional network modifier is that it has a low charge density when compared to Mg. Charge density expressed as cation field strength (CFS) is 0.46 Å^–2^ for Mg, but 0.18 Å^–2^ for Na [23]. The CFS of a network-modifier can have a significant effect on the structure of aluminosilicate glasses [24]. Hence, Na-Mg aluminosilicate glasses are interesting systems for understanding the effect of CFS on structure. To allow a wider use of Na-Mg aluminosilicate glasses for AAM production, in-depth studies on their reactivity must be conducted. Reactivity is a necessary, although not sufficient, requirement for a suitable precursor for AAMs. One important parameter controlling their reactivity is their structure.

**Fig 1:**
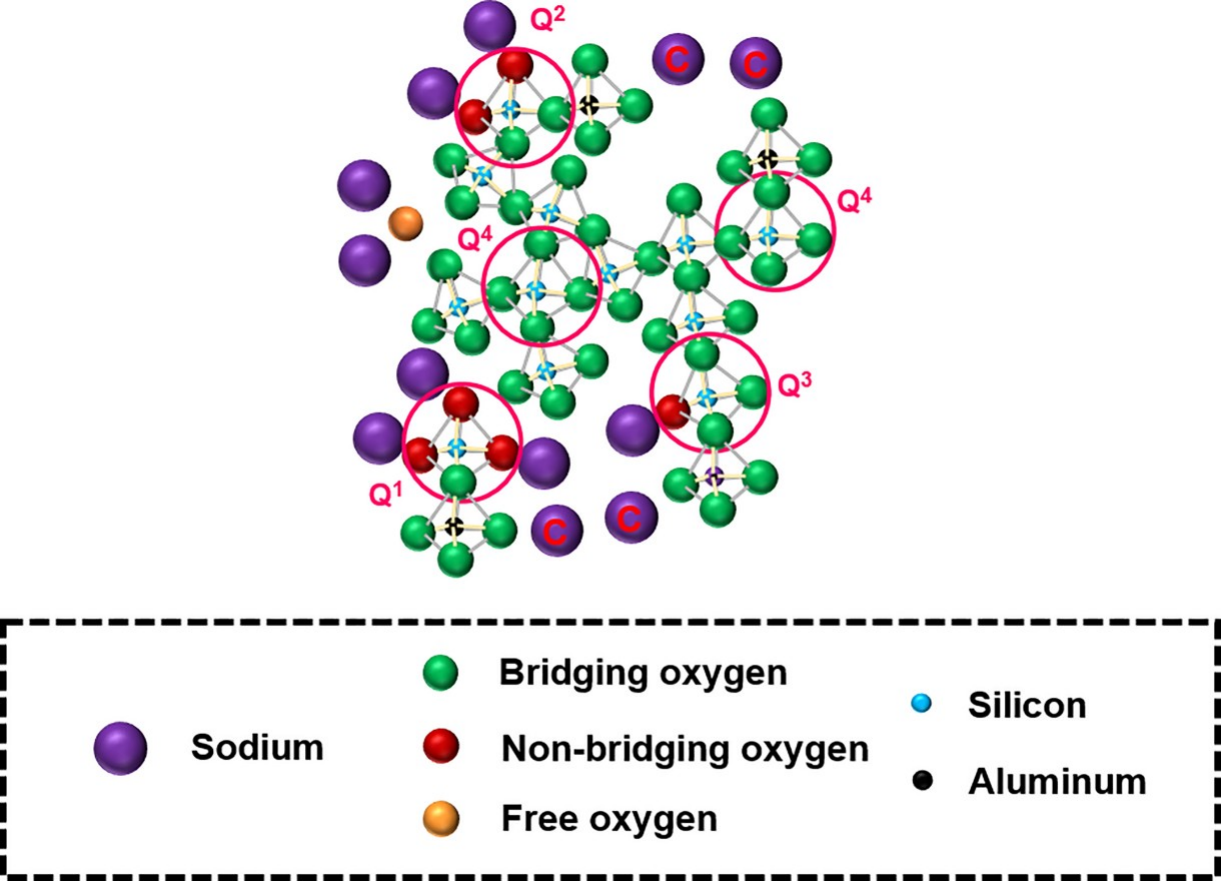
Simplified structure of a ternary sodium-aluminosilicate glass. Roles of Na are as follows: those acting as charge compensators are labeled “C;” those in the immediate vicinity of non-bridging oxygen atoms act as network modifiers; those in the immediate vicinity of free oxygen exist as free oxide. The Si species are depicted in Q^n^ form, where n (0 ≤ n ≤ 4) represents the number of bridging oxygen atoms around the silicon atom as the nearest neighbors.

The structure of an aluminosilicate glass generally consists of a 3D network of SiO_4_ and AlO_4_ tetrahedra with bridging oxygen to allow the polymerization of the network [25–28]. Network-modifying cations (such as alkali or alkaline earth metals) disrupt the network by forming non-bridging oxygen, so they contribute to the depolymerization of the network. Network-modifying cations can also perform the role of charge compensation to neutralize the negative charge carried by AlO_4_ tetrahedra, thereby contributing to the polymerization of the network. When network-modifying cations are present in excess quantities, a portion of them can exist in free oxide form, which contributes to neither the polymerization nor the depolymerization of the network [29]. The simplified structure of a ternary sodium-aluminosilicate glass is shown in Fig 1.

One of the most commonly used parameters to represent the reactivity of aluminosilicate glasses is the number of non-bridging oxygens (NBO) per tetrahedral cation (T), or NBO/T [30,31]:

$$\frac{NBO}{T}=\frac{2\left(X_{MO}+X_{M_{2}O}+3f_{M_{2}O_{3}}-X_{Al_{2}O_{3}}-(1-f)X_{M_{2}O_{3}}\right)}{X_{SiO_{2}}+2X_{Al_{2}O_{3}}+2(1-f)X_{M_{2}O_{3}}}$$ (1)

where X = mole fraction; MO = CaO, MgO, FeO, etc.; M_2_O = Na_2_O, K_2_O, etc.; M_2_O_3_ = Al_2_O_3_, Fe_2_O_3_, Cr_2_O_3_, etc., and *f* = fraction of M_2_O_3_ acting as a network modifier. In general, the higher the NBO/T, the higher the reactivity of the glass. One disadvantage of using NBO/T as a sole parameter is that it does not differentiate between the effects of different network-modifying cations. Hence, the concept of optical basicity (Λ) is used, as it takes into account the effects of different network-modifying cations [30,32–35].

$$\Lambda =\frac{\Sigma X_{i}n_{i}\Lambda_{i}}{\Sigma X_{i}n_{i}}$$ (2)

where *X*_*i*_ = mole fraction of the component; *n*_*i*_ is the number of oxygen atoms in the formula of the component (e.g.: *n*_*i*_ = 3 for Al_2_O_3_, while n = 2 for SiO_2_), and is the optical basicity value of the component (e.g.: *Λ*_*i* =_ 0.78 for MgO, while *Λ*_*i* =_ 1.15 for Na_2_O) [30]. The higher the optical basicity, the higher the reactivity of the glass.

In this work, synthesis, characterization, and alkaline solubility of Na-Mg aluminosilicate glasses were carried out with the main purpose of understanding the role of network-modifying cations. A series of 10 quaternary aluminosilicate glasses ((Na_2_O)_1-x_(MgO)_x_ (Al_2_O_3_)_0.25_(SiO_2_)_1.25_; 1 ≥ x ≥ 0) are synthesized. Note that all glasses have similar Si/Al ratios and similar theoretical NBO/Ts (0.857), while the optical basicity (Λ) decreases from 0.659 to 0.572 on moving from Na endmember to Mg endmember. The synthesized glasses are characterized by time-gated Raman spectroscopy, Si K-edge X-ray absorption near edge spectroscopy (XANES), and nitrogen physisorption. Alkaline reactivity of glasses was estimated using a concentrated sodium hydroxide solution. Solid residues after the solubility test were characterized by X-ray diffraction (XRD) and scanning transmission electron microscopy (STEM) to obtain more insights into the alkaline dissolution of the glasses.

## 2. Materials and methods

### 2.1 Materials

Preparation of Na-Mg aluminosilicate glasses involved the following precursors: sodium carbonate (Sigma-Aldrich), magnesium oxide (Sigma-Aldrich), silicon oxide (Alfa Aesar), and aluminum oxide (Sigma-Aldrich). For the alkaline solubility testing of glasses, sodium hydroxide (purity > 99%) obtained from Merck (Germany) was used. Nitric acid (64–66%) obtained from Sigma-Aldrich was used to neutralize the filtered alkaline solution. Deionized water was used wherever required.

### 2.2 Glass preparation

Glass preparation involved 10 Na-Mg aluminosilicate glasses with fixed compositions ((Na_2_O)_1-x_(MgO)_x_(Al_2_O_3_)_0.25_ (SiO_2_)_1.25_; 1 ≥ x ≥ 0). Prepared glasses were represented as Gx for simplicity. The glass precursors were mixed in the desired proportions and ground in a vibratory disc mill (Retsch RS 200) at a speed of 1500 rpm for 3 minutes. Each batch consisted of 50 g of mixture. The mixture was transferred to a platinum crucible, which was placed into a Nabertherm high-temperature furnace (HT 08/18). The mixture was heated from room temperature at 20°C/min to 1600°C, then held for 90 minutes. At the end of the holding time, the crucible was removed from the furnace, and the melt was rapidly quenched by pouring it into water at room temperature. The glass pieces were collected and subsequently dried in an oven at 60°C for 48 hours. The dried glass pieces were subjected to a second melting process using the same glass making procedure as before). The dried glass pieces were milled in a vibratory disc mill (Retsch RS 200) at a speed of 1000 rpm for 1–5 minutes to obtain an average particle size between 1 μm and 10 μm before further characterization and solubility testing.

Note the following: 1) A melting temperature of 1600°C was chosen to ensure complete melting of the precursors. In practical situations, the melting temperature could be lowered by using fluxes; 2) The second melting process was carried out to ensure homogeneity of the prepared glasses [22,36].

### 2.3 Solubility test

The solubility test was a modified version of the one reported earlier in the literature [37]. Each powdered glass sample was mixed with 6M NaOH solution in a polypropylene bottle with a liquid-to-solid weight ratio of 40. The test was performed for 24 hours at 23 ± 0.5°C under a shaking motion (2.5 Hz) using a horizontal shaking table (IKA KS 260 orbital shaker). At the end of the solubility test, the sample was filtered using a 0.45 μm nylon filter paper. The filtrate was acidified with nitric acid to pH less than 2 (to prevent precipitation of dissolved species), and it was later analyzed by ICP-OES to determine elemental concentrations. The solid residue was rinsed with deionized water multiple times and then dried in a desiccator for 2 days.

### 2.4 Characterization techniques

X-ray diffraction (XRD) patterns of samples were recorded using a Rigaku SmartLab 9 kW XRD machine. The analysis involved the following parameters: Co Kα radiation (Kα1 = 1.78892 Å; Kα2 = 1.79278 Å; Kα1/Kα2 = 0.5), a scan rate of 3°/min between 5°and 85° 2θ, and 0.02°/step size. For phase identification, “X’pert HighScore Plus” (PANalytical software) was used. Raman spectroscopic analysis was performed using a time-gated Raman spectrometer (PicoRaman and model M1 devices, Timegate Instruments Inc.) fitted with a fiber-coupled pulsed (532 nm) laser and a single photon counting CMOS SPAD matrix detector with ~5 cm^-1^ spectral resolution. The data were collected in the Raman shift range from 300 cm^-1^ to 1200 cm^-1^. The data treatment involved the use of the AirPLS method for background correction. Raman spectra were deconvoluted into Gaussian components with the help of Origin software. During deconvolution, peak position, intensity, and FWHM (full width at half maximum) were allowed to vary independently while the χ^2^ minimization principle was followed. (More details of peak position and FWHM are provided in the supporting information, S1 Table).

The specific surface areas of the samples were determined by physisorption of nitrogen at −196°C using a Micromeritics ASAP 2020 instrument. The specific surface areas were obtained using the Brunauer-Emmett-Teller (BET) method [38]. Prior to nitrogen physisiorption, the samples were degassed under vacuum at 100°C for 2 hours. Scanning transmission electron microscope (STEM) imaging was performed using a JEOL JEM-2200FS equipped with energy-dispersive X-ray spectroscopy (EDS) detector (JEOL Dry SD100GV, 100 mm^2^, 0.98 Sr).

Elemental concentrations in the liquid samples were analyzed using an inductively coupled plasma optical emission spectrometer (ICP-OES; Thermo Fisher Scientific iCAP6500 Duo) fitted with a Cetac ASX-520 auto sampler.

Si K-edge X-ray absorption measurements were performed at the SXRMB beamline of Canadian Light Source, Saskatoon, Canada. Powder samples were mounted onto double-sided, conductive carbon tape and loaded into the vacuum chamber. Si wafer and SiO_2_ were used as references and for energy calibration. A 7-element SDD detector was used to record the fluorescence spectrum of the powder samples. For samples with high Si concentration and Si references, the total electron yield (by recording the drain current off the sample) was used to avoid the self-absorption problem associated with the fluorescence mode. The Athena program was used for data analyses, including background removal, normalization, and EXAFS analysis [39].

## 3. Results and discussion

### 3.1. Characterization of synthetic glasses

Raman spectroscopy is often used for structural analysis of aluminosilicate glasses. Raman spectra of aluminosilicate glasses are traditionally divided into 3 regions: a low-frequency region (300–700 cm^-1^); an intermediate-frequency region (700–850 cm^-1^); and a high-frequency region (850–1300 cm^-1^). The low-frequency region carries information about ring sizes and tetrahedral cations, while the medium-frequency region contains several overlapping bands and is usually not used for analysis [27]. The high-frequency region is the one used the most; it contains information about the concentration of non-bridging oxygen and the Al/Si mixing [27].

**Fig 2:**
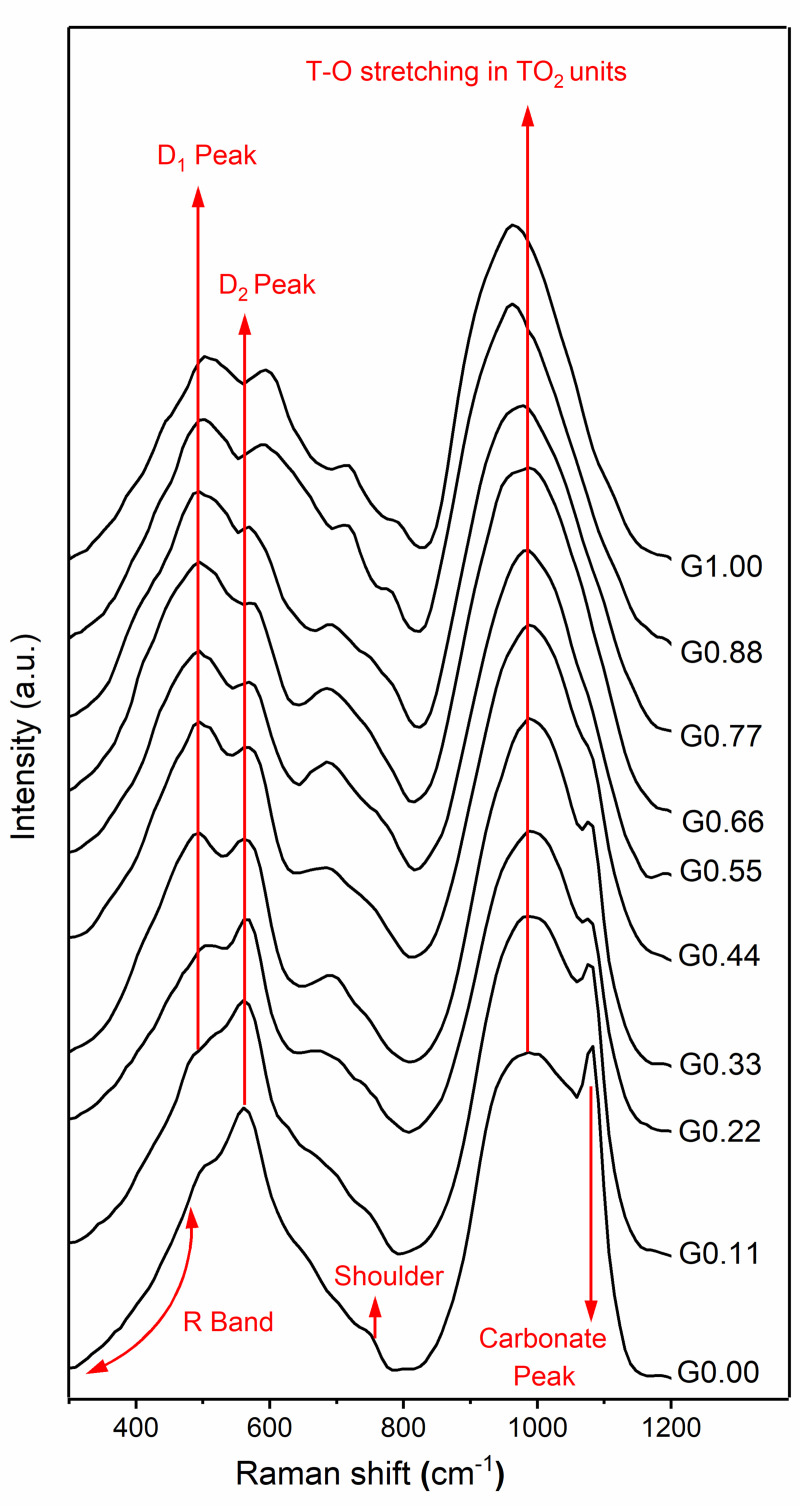
Raman spectra of Na-Mg aluminosilicate glasses. The value of x in the notation Gx reflects the MgO content as defined in section 2.2.

The low frequency region of the spectra for the synthetic glasses studied here contained three main features: R band, D_1_ peak, and D_2_ peak (Fig 2). The R band (300–470 cm^-1^) is related to the breathing motion of oxygen (the movement of oxygen atoms perpendicular to Si-O-Si plane) in five-, six-, or higher-membered tetrahedral rings [26]. The D_1_ peak (490–500 cm^-1^) is attributed to the breathing motion of oxygen atoms in four-membered rings, while the D_2_ peak (560–600 cm^-1^) concerns the breathing motion of oxygen atoms in three-membered rings [40–42]. Another band starts growing around 690 cm^-1^ in the Na endmembers, then it gradually increases its frequency until it reaches around 710 cm^-1^ in the Mg endmember. The origin of this band is unknown.

The mid-frequency region is relatively featureless when compared to other regions. In the case of the Na endmember, there is a shoulder visible around 760 cm^-1^ and this shifts around 800 cm^-1^ toward the Mg endmember. A similar trend has been reported when Ca substitutes for Na in Na/Ca silicate glasses [43] as well as when the Sr concentration increases in Na/Sr silicate glasses [44]. This band could be attributed to Si–O stretching, involving oxygen motion, or to the motion of silicon atoms in the oxygen cage [27,29,45].

The high-frequency region exhibits a prominent band from 800 cm^-1^to 1200 cm^-1^. This band has been attributed to T-O stretching in TO_2_ units, where T = Si or Al [26]. In addition, the Na endmember shows a carbonate peak around 1085 cm^-1^. This comes from sodium carbonate, which is formed because of atmospheric carbonation of free oxides of sodium present in the glasses. This band almost disappears from glasses after G0.44.

**Fig 3:**
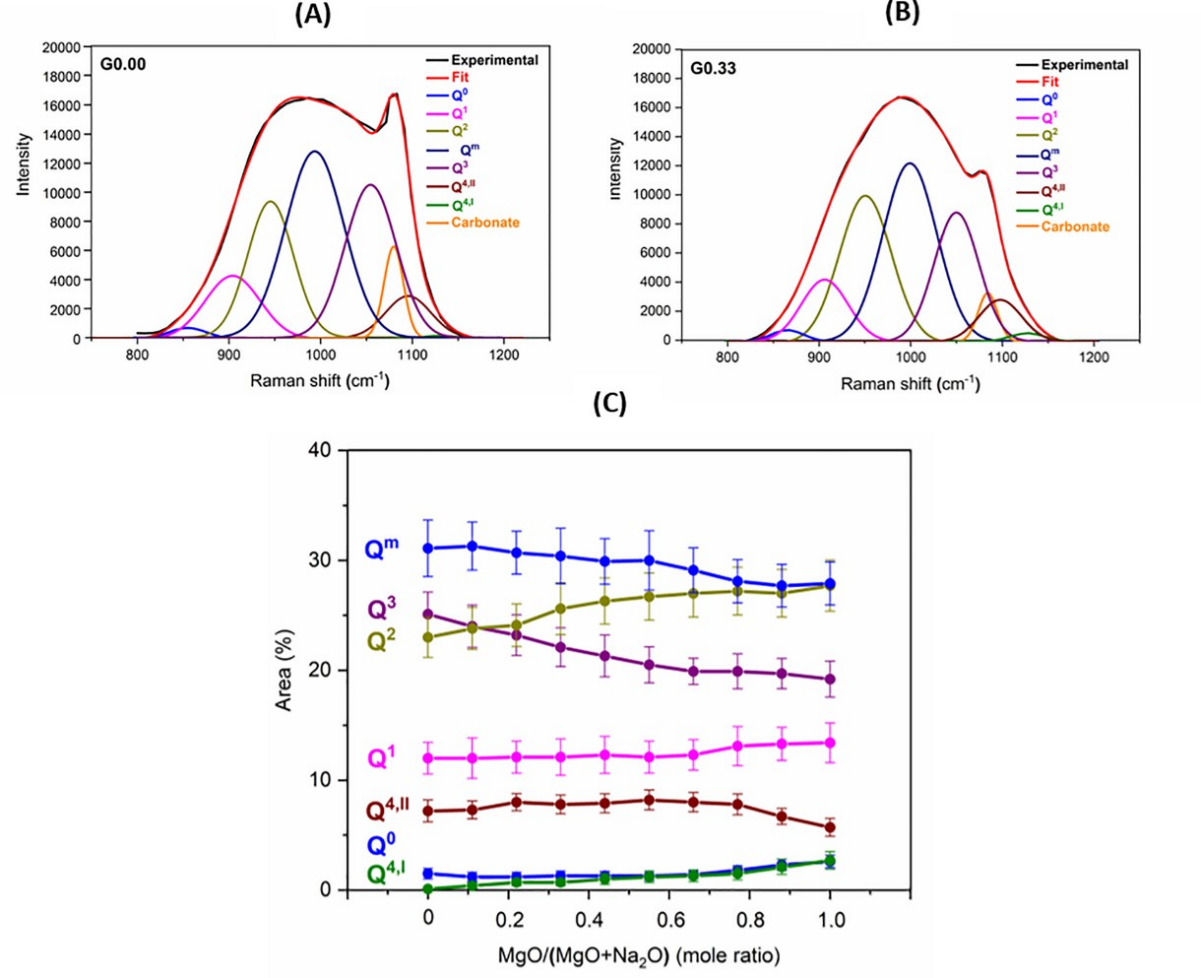
(A) Deconvolution of Raman spectra for glasses G0.00. (B) Deconvolution of Raman spectra for glasses G0.33. (C) Q^n^ distribution plot for glasses.

The high-frequency region of the Raman spectra can be deconvoluted to provide information about the Q^n^ species present in the glasses. However, two important things should be noted: 1) Raman spectroscopy does not differentiate contributions from SiO_4_ and AlO_4_ tetrahedra [46]; 2) The deconvolution does not provide true concentrations of Q^n^ species, as the intensity of the components depends not only on the concentration but also on their local environment [47]. However, deconvolution can be used for describing variations in relative proportions of various Q^n^ species [48]. In our work, the spectra have been deconvoluted into Gaussian components representing various species (Fig 3A and 3B). The positions of the components were decided after consulting literature [29,47,49–52], and were as follows: Q^0^ (860 ± 3) cm^-1^), Q^1^ ((900 ± 5) cm^-1^), Q^2^ ((950 ± 3) cm^-1^), Q^mixed^ ((1000 ± 3) cm^-1^), Q^3^ ((1050 ± 4) cm^-1^), Q^4,II^ (1100 ± 3) cm^-1^), Q^4,I^ ((1130 ± 3) cm^-1^), and the carbonate component (1085 ± 3) cm^-1^). The Q^m^ component is complicated, and it has been attributed to the vibrations of bridging oxygen in structural units which need not be completely polymerized [29]. Q^4,II^ species are associated with T-O-T linkages whose bond angle is smaller when compared to those of Q^4,I^ species [46,53]. This means Q^4,II^ is more related to Si-O-Al, while Q^4,I^ is concerned with Si-O-Si. The deconvolution of glasses is shown in Fig 3A and 3B.

The results of the deconvolution (Fig 3C) indicate that as Mg was introduced in the Na endmember, there was a decrease in the proportion of the Q^3^ species and an increase in the proportion of the Q^2^ species. Towards the Mg endmember, there were some minor changes which included a decrease in the proportions of Q^4,II^ and an increase in the proportions of Q^4,I^ species. This indicated a decrease in the proportion of aluminum-rich Q^4^ species and an increase in the proportion of aluminum-poor Q^4^ species. There was also a slight increase in the proportion of highly depolymerized species (Q^0^ and Q^1^) toward the magnesium endmember.

The trend in relative Q^n^ speciation derived from Raman spectroscopy analysis agrees with the results of Q^n^ speciation obtained by authors through deconvolution of ^29^Si MAS NMR spectra for the same series of Na-Mg aluminosilicate glasses [24]. It has been found that replacing Na with Mg in aluminosilicate glasses leads to a reduction in the amount of Q^3^(0Al), Q^3^(1Al), and Q^3^(2Al) species, while the amount of Q^2^(0Al) and Q^2^(1Al) increases. It has been observed that towards the Mg endmember, there is a reduction in the amount of aluminum-rich Q^4^ species (Q^4^(4Al) and Q^4^(3Al)), while the amount of aluminum-deficient Q^4^ species (Q^4^(2Al) and Q^4^(1Al)) increases. A slight increase in the amount of Q^1^ and Q^0^ species has been observed towards the Mg endmember.

Previous structural studies on glasses using techniques like Raman spectroscopy, ^27^Al and ^29^Si MAS, and MQMAS spectroscopies indicate the preferential association of alkaline earth metals with the Q^2^ species, while alkali metals show more affinity for the Q^3^ species [23,43,54–57]. According to the structural study of aluminosilicate glasses using ^29^Si MAS NMR spectroscopy, alkali metal as charge compensator leads to an increase in the proportions of Q^4^(4Al) and Q^4^(3Al) species (when compared to other Q^4^(mAl) species), while an alkaline earth metal as the charge compensator results in an increased proportion of Q^4^(2Al) and Q^4^(1Al) species (when compared to other Q^4^(mAl) species) [48,58]. It has been reported that cations with higher charge density, such as Mg, have a greater tendency to form wider distribution of Q^n^ species (i.e., formation of highly polymerized species (like Q^4,I^) as well as formation of highly depolymerized species (like Q^0^, Q^1^) [30,59].

**Fig 4:**
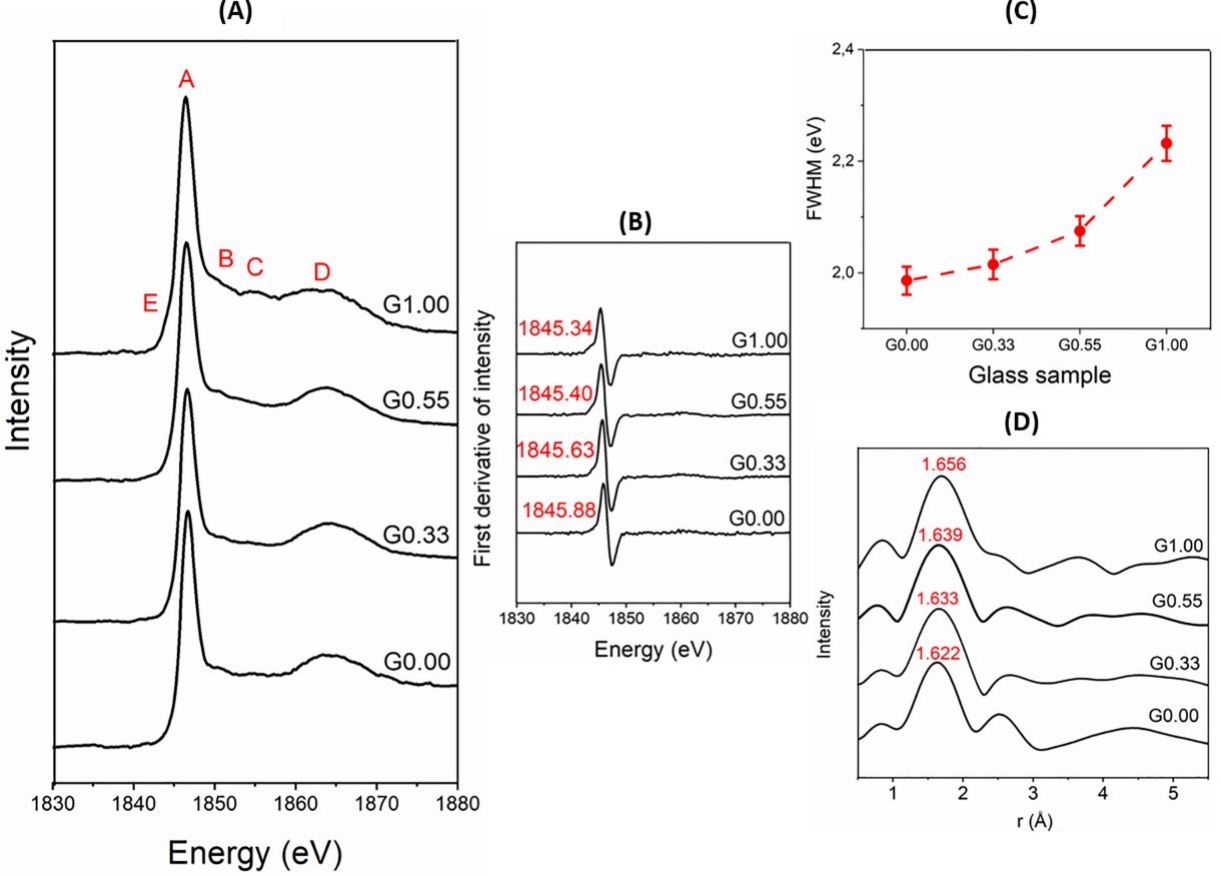
(A) XANES spectra at Si K-edge of Na-Mg aluminosilicate glasses; (B) first derivative of XANES spectra, with the energy corresponding to the maximum in the derivative marked for each sample; (C) FWHM plot for Si K-edge.; (D) Fourier transform (radial distance) of Si-Kedge EXAFS spectra.

Extended X-ray absorption fine structure (XANES) can be used to study the local order around silicon atoms in aluminosilicate glasses. Si K-edge XANES spectra of Na-Mg aluminosilicate glasses (Fig 4A) revealed five main peaks; peak A (≈ 1846eV) is attributed to the Si 1s → t_2_ states (Si 3p/3s) transition; peak B (≈ 1851eV) is produced by multiple scattering; peak C (≈ 1855eV) corresponds to the Si 1s → e states (Si 3d/3p) transition; peak D (≈ 1864eV) indicates the Si 1s → to t_2_ states (Si 3d/3p) transition; and peak E (≈ 1844eV) is attributed to Si 1s → to a_1_ states (Si 3s/3p) transition. The energy positions of the observed peaks are in agreement with those reported in the literature [60–63].

Peak A is the X-ray white line. The K-edge occurs below this peak to the side of low-energy [63]. The energy position of peak A indicates that silicon exists in tetrahedral coordination in the glasses [64]. Peak B is more pronounced in the Mg endmember when compared to other glasses. It has been reported that peak B varies in intensity according to the type of network-modifying cation and it is more intense in Ca silicate glasses than in Na silicate glasses [63]. Peak C is more intense in the Mg endmember when compared to other glasses. A previous study found that peak C is predominant in Ca silicate glasses when compared to Na/K silicate glasses [63], so it appears to relate mainly to the structures that include divalent cations. Peak D is a relatively broad feature; changes in this peak have been correlated to the average Si-Si bond length and Si-O-Si bond angle [65,66]. Hence, broadening this peak (especially in the Mg endmember) with the addition of Mg implies a wider distribution in bond length and bond angle. This is in agreement with the Raman spectral analysis presented above, which indicated an increase in the proportion of Q^4,I^ sites toward the Mg endmember. (The Mg endmember has considerable amounts of both Q^4,I^ and Q^4,II^ sites, while the Na endmember has mostly Q^4,II^ sites.) There is also a shift of peak D to lower energy as the Mg content of glasses increases. A similar trend has been observed when Ca replaces Na in silicate glasses [63]. Peak E is more pronounced only in the Mg endmember. The prominence of this band could be because of bond mixing of Si 3s and 3p orbitals [62].

The energy position of the Si K-edge and the FWHM of the white line K-edge are shown in Fig 4B and 4C. The K-edge position has been obtained by finding the local maxima in the first derivative of the intensity with respect to energy. There is a slight decrease in the K-edge on moving from G.00 to G1.00. The K-edge position can be regarded as an indicator of Q^n^ species, and it shifts to lower energies on moving from Q^4^ to Q^0^ species [63,67]. Hence, the decrease in K-edge position indicates increased depolymerization in glasses as Na is replaced with Mg. This comports with the Raman spectral analysis. The FWHM of the K-edge increases gradually when moving from G0.00 to G1.00. That indicates increased structural disorder toward the Mg endmember. This agrees with wider distribution of Q^n^ species toward the Mg endmember, as indicated by deconvolution of the Raman spectra of glasses.

The EXAFS radial distance plot calculated from these data is shown in Fig 4D. The first and strongest shell is contributed mostly by the Si-O correlation (≈1.6Å), and partly by the Al-O correlation (≈1.7 Å). It is also possible that a minor contribution comes from Mg-O correlations (2.0 to 2.3Å). The position of this shell increases slightly when moving from G0.00 to G1.00. This could be because of increased depolymerization. The width of the first peak also increases when moving from G0.00 to G1.00. This could reflect a wider distribution of bond lengths and increased disorder. Since Si is a weak scatterer (as it is a light element), it is difficult to get reliable information beyond the first shell.

### 3.2 Alkaline reactivity of synthetic glasses

Alkaline reactivity of glasses has been estimated by analyzing their solubility in 6M NaOH solution. The reactivity results are expressed as the percentage of specific element dissolved, based on its original content in the glass before dissolution. Nitrogen physisorption measurements were taken to estimate the surface areas of the synthetic glasses. Glasses are found to have a low surface area of around 1 m^2^/g. Detailed results are provided in the supporting information, S2 Table. The calculated solubility is normalized with respect to the surface area of G0.00 to negate the effect of surface area contributing to the solubility (refer to supporting information, S3).

**Fig 5:**
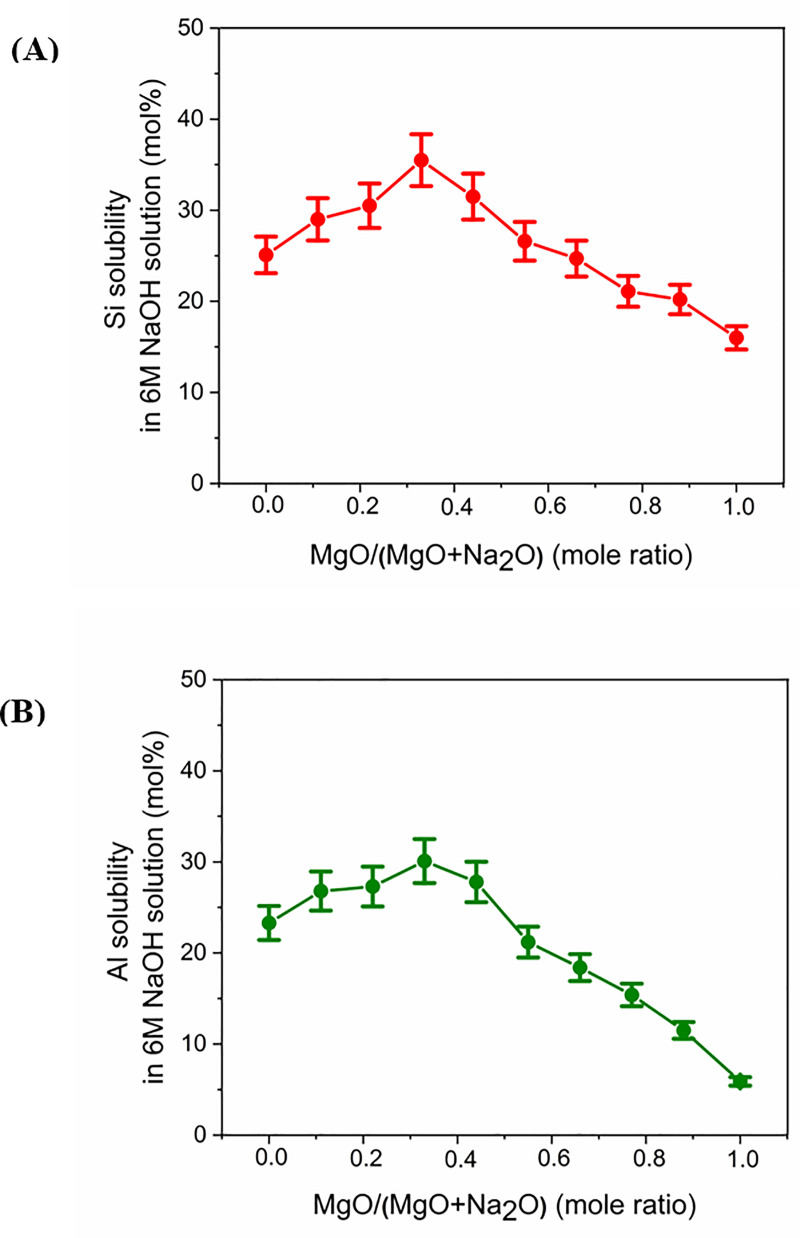
Results of alkaline solubility of (A) silicon and (B) aluminum. Results are expressed as the weight percentage of the respective elements that dissolved during the solubility tests, relative to their amounts in the original glasses.

Data for the alkaline solubility of silicon (Fig 5A) indicate that the Na endmember (*x* = 0, where *x* = MgO/(MgO+Na_2_O), expressed as mole fraction) showed Si solubility around 25 mol %. As the value of *x* increases from 0, there is a gradual rise in solubility until *x* = 0.33. The glass with *x* = 0.33 showed the highest solubility of around 35%. With further increases in *x*, the solubility gradually drops. The lowest silicon solubility (around 16%) is observed for the Mg endmember (*x* = 1).

In case of Al solubility (Fig 5B), the Na endmember (*x* = 0) exhibited solubility of around 23%. As the value of *x* rises from 0, there is a gradual increase in solubility until *x* = 0.33. This trend is similar to the Si solubility trend. However, the proportional increase is less when compared to that of Si. The glass with *x* = 0.33 showed the highest solubility—around 30%. With a further increase in *x*, the solubility drops. This also is similar to the silicon solubility trend. However, the proportional decrease is higher than that of Si, and the Al solubility reached its lowest value of around 6% in the Mg endmember. The reasons for the differences in Al solubility will be explored in detail below. It is not expected that dissolution would be incongruent under such far-from-equilibrium conditions, and so it is likely that this behavior is being driven by reprecipitation reactions.

**Fig 6:**
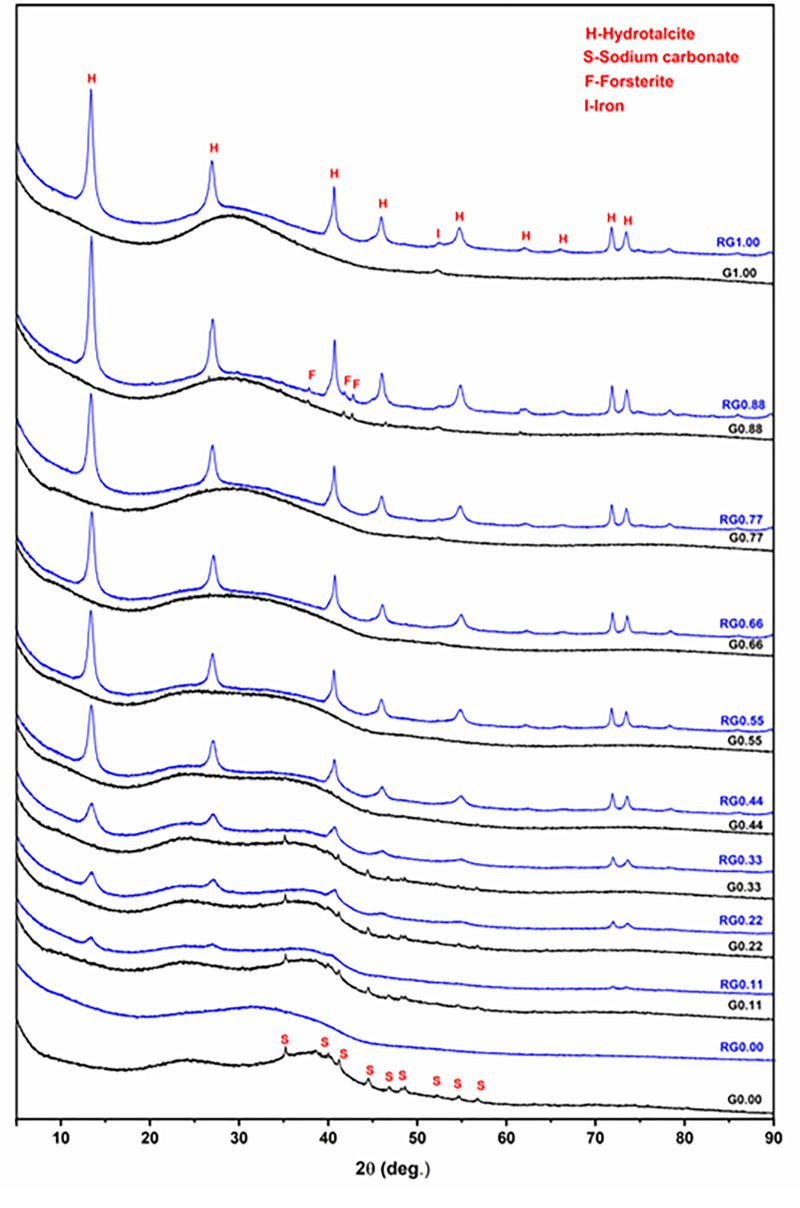
XRD results for the glasses (labeled as Gx) and the solid residues (labeled as RGx) remaining after their alkaline solubility tests.

XRD analysis of the solid residue (remaining after the alkaline solubility test) was performed to gain further insights into the alkaline dissolution of these glasses (Fig 6). For the purpose of comparison, the XRD patterns of the original glasses also are reported. The Na endmember (G1.00) showed largely amorphous features except for crystalline contributions from a minor sodium carbonate phase, which is formed by atmospheric carbonation of the free form of Na (sodium oxide). The solid residue (RG0.00) is completely amorphous, and it does not show the crystalline sodium carbonate component, implying that it dissolved during the alkaline dissolution process. This trend is repeated in G0.11, G0.22, and G0.33. In the case of G0.11, the XRD pattern is similar to that of G0.00. The solid residue (RG0.11) is largely amorphous except for the crystalline hydrotalcite component. This means that a part of the dissolved Al precipitated together with Mg as hydrotalcite. The trend in the XRD analysis of the solid residue of glasses from G0.22 to G1.00 is similar to that of G0.11, except that hydrotalcite grows more intense toward the Mg endmember, as more Mg is available for hydrotalcite formation. This explains the steep decrease in solubility of Al in comparison to Si shown in Fig 5. One of the glasses toward the Mg endmember, G0.88, showed a very minor crystalline phase of forsterite (Mg_2_SiO_4_). The solid residue (RG0.88) retained this component, which indicates that forsterite is inert to alkali attack under the test conditions. A small, fairly broad peak is visible around 52.3^o^ 2θ in the diffractograms of G0.77, G0.88, and G1.00. This corresponds to metallic Fe, which originates from the steel grinding equipment used to pulverize the glass samples.

**Fig 7:**
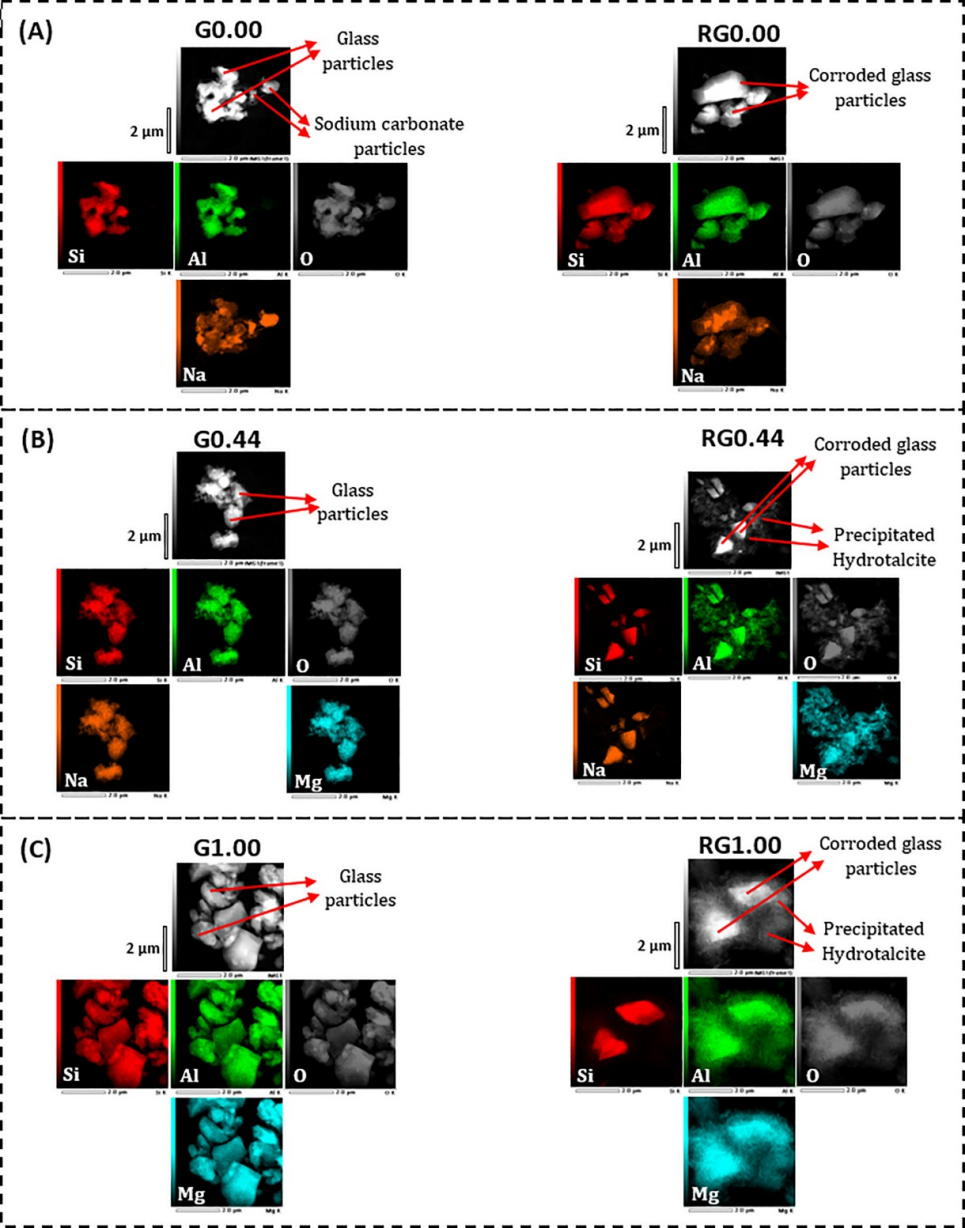
STEM dark field images along with elemental mapping for the glasses (labeled Gx) and the solid residues (labeled RGx) remaining after their alkaline solubility tests.

The STEM dark field images along with elemental mapping have been obtained for both the glasses and the solid residues that remained after the alkaline solubility tests (Fig 7). The Na endmember (G0.00) consisted of irregularly shaped particles with uniform distributions of Si and Al. However, the distribution of Na was not uniform, and the regions of its high concentration indicated the presence of sodium carbonate. This agrees with the XRD results (Fig 6). The solid residue (RG0.00) consisted of smoothened corroded glass particles without any observable precipitated components. It also indicated regions of high sodium concentration, which may correspond to sodium carbonate, although XRD analysis did not detect this phase in RG0.00 (Fig 6). The glass sample G0.44 had irregularly shaped particles with uniform distributions of Si, Al, and Na. After alkali dissolution, the corresponding solid residue (RG0.44) showed corroded glass particles along with precipitated scale-like features surrounding them. These precipitated components are rich in Al and Mg and devoid of both Si and Na. This result is consistent with the XRD analysis, which observed crystalline hydrotalcite formation after alkaline dissolution. The trend found in the G0.44 to RG0.44 transformation is similar to that of the G1.00 to RG1.00 transformation, except that the precipitated hydrotalcite phase is more intense in the latter. This is in agreement with the XRD analysis, which indicated higher growth of hydrotalcite toward the magnesium endmember. In short, the microscopic analysis is in agreement with the XRD analysis as well the results of Si and Al alkaline solubility.

### 3.3 Further discussion of alkaline reactivity of synthetic glasses

Results of both the XRD and STEM analysis of the solid residues after alkaline solubility test indicated that Al contributed to the precipitate formation. Hence, Al solubility cannot be taken to represent the alkaline reactivity of the glasses. On the contrary, Si did not visibly contribute to the precipitate formation. Hence, Si solubility can used to estimate the alkaline reactivity of the glasses under the conditions of the test applied here.

**Fig 8:**
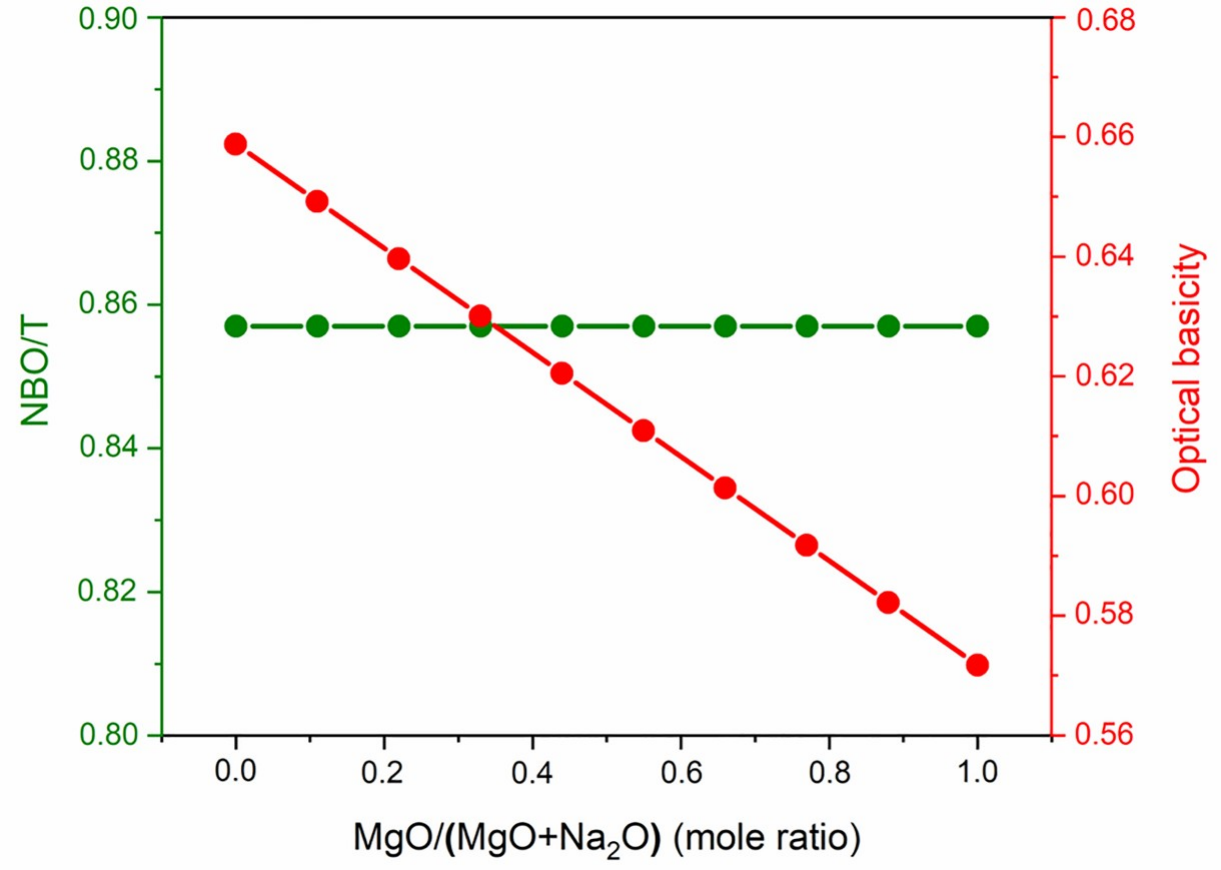
NBO/T and optical basicity of glasses.

The Si solubility study indicated that glass reactivity rises initially as the Mg content of the glasses increases. It reaches a maximum for *x* = 0.33, after which the reactivity falls. This trend in reactivity cannot be explained by either of the conventionally used parameters for glass reactivity—NBO/T and optical basicity. NBO/T and optical basicity values for all the glasses are plotted in Fig 8. NBO/T values for glasses were calculated using Eq 1. All the glasses were found to have same theoretical NBO/T (0.857). Optical basicity values of glasses were calculated using Eq 2. The optical basicity value of the Naendmember was 0.659. With the addition of Mg into the glasses, the optical basicity decreased and reached its lowest value (0.572) in the Mg endmember. As all the glasses had the same NBO/T, they should show similar reactivity, but that was not consistent with the experimental results. Another drawback of NBO/T as a parameter is directly indicated by the XRD analysis of G0.88 and RG0.88. Forsterite (Mg_2_SiO_4_) is present as a minor phase in both G0.88 and RG0.88, and it is highly inert toward alkaline attack. However, the NBO/T value of forsterite is 4 (as a nesosilicate mineral). This is the highest possible value for a silicate, so it should be highly reactive according to this formalism. The inertness of forsterite contradicts the prediction based solely on NBO/T as a parameter. The other parameter for glass reactivity, optical basicity, cannot explain the glass reactivity completely. The optical basicity of glasses decreases as Na is replaced with Mg in the glasses. This means that the reactivity of glasses should decrease with the addition of Mg. This can probably explain the decline in glass reactivity after the optimal reactivity. However, it fails to explain the reason for the initial increase in glass reactivity.

**Fig 9:**
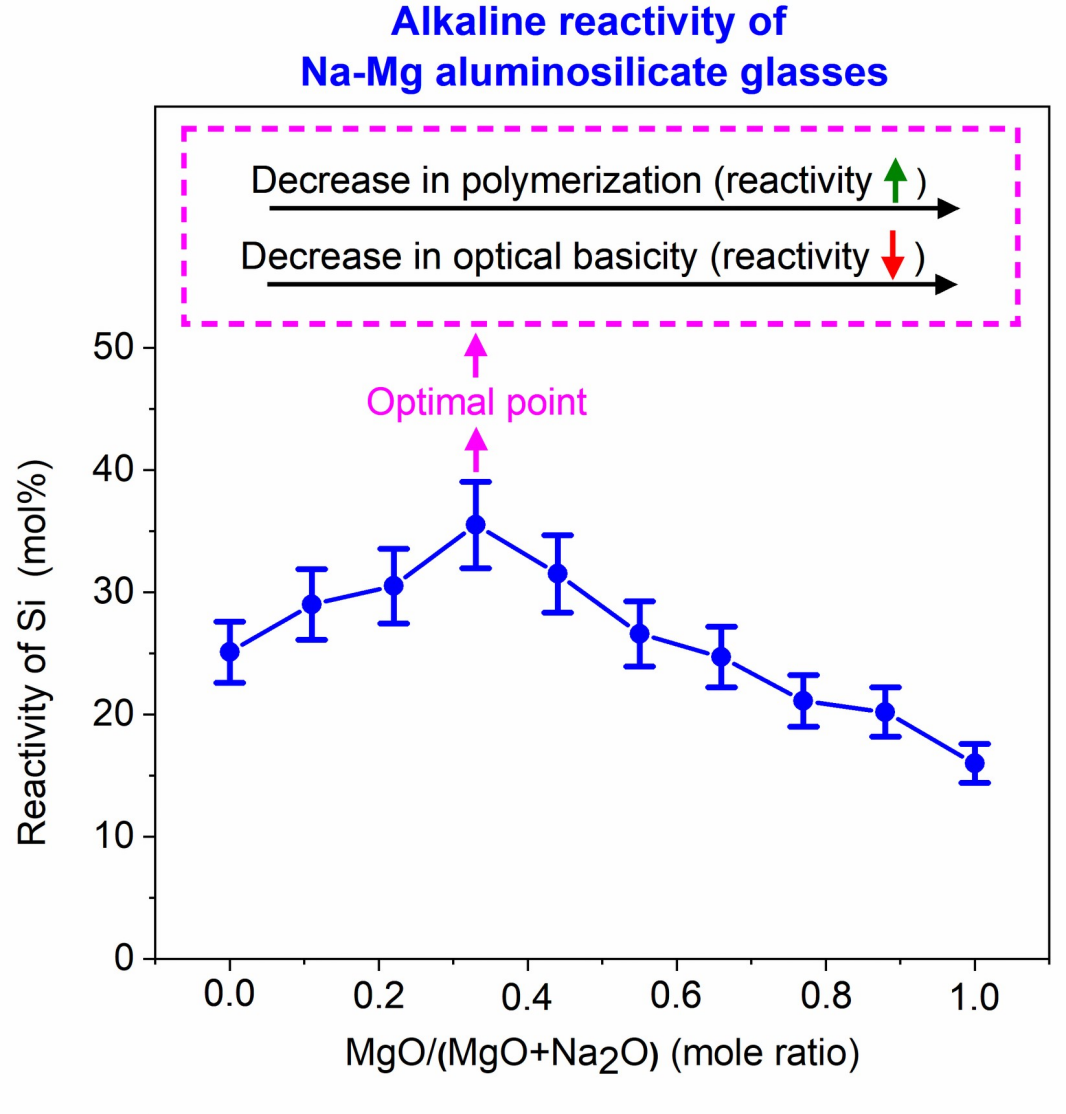
Influence of polymerization and optical basicity on reactivity of Na-Mg aluminosilicate glasses.

Understanding the alkaline reactivity of the glasses needs due consideration of their structure apart from the conventional parameters like optical basicity and NBO/T. The structural study of glasses using Raman spectroscopy indicated that there was increased depolymerization in the glasses as Mg replaced Na in aluminosilicate glasses. (Most notable was the increase in the proportion of the Q^2^ species and the decrease in the proportion of the Q^3^ species.) A more depolymerized glass would show higher reactivity. The alkaline reactivity of the aluminosilicate glasses studied was dominated by two factors: 1) depolymerization of the glasses; 2) optical basicity. When smaller amounts of Mg replaced Na, the effect of depolymerization dominated that of optical basicity. This led to an increase in reactivity as Na replaced Mg in the glasses. However, at higher Mg contents, the effect of optical basicity dominated depolymerization, so the glass reactivity decreased. These results are summarized in Fig 9.

The alkaline reactivity of the glasses studied in this work specifically concerns how chemical durability (higher chemical durability implies lower alkaline reactivity) was influenced when one network modifier was substituted for another while keeping the equivalent network modifier content the same. The substitution of network modifier in silicate glasses is known to cause non-linear variation of many physical properties, and this has been attributed to what is known as the mixed-modifier cation effect (MMCE) [68]. There have been controversial observations in the studies of the influence of MMCE on the chemical durability of silicate glasses. Bunker et al. [69] and Smets et al. [70] concluded that there is no evident influence of MMCE on the chemical durability of silicate glasses. However, Day et al. [71] and Zhifang et al. [72] concluded that MMCE did influence the chemical durability of glasses. According to the latter, replacing one network modifier with another led to an increase in the chemical durability in the initial stages, but the chemical durability decreased in the later stages. However, that observation contradicts our result that as Mg replaced Na in aluminosilicate glasses, chemical durability decreased initially, but then it increased during the later stages. However, most of the studies have been done on silicates systems with no or low Al content. It has been reported that the influence of MMCE on chemical durability disappeared in Na/K/Ca silicate glasses when a small fraction of Si was substituted by Al [72]. The conflicts in the conclusions about chemical durability in the literature could be because the researchers did not take into account detailed structural study of glasses and parameters like optical basicity.

## 4. Conclusions

Raman spectra of Na-Mg aluminosilicate glasses showed typical features of aluminosilicate glasses: R band, D_1_ peak, D_2_ peak, and the prominent high frequency band stretching from 800 cm^-1^ to 1200 cm^-1^. Deconvolution of the high-frequency band yielded information about the Q^n^ distribution. The major change happening as Mg replaced Na in aluminosilicate glasses was the reduction in the Q^3^ species and the increase in the Q^2^ species. Toward the Mg endmember, there was a reduction in the proportion of Q^4,II^ and an increase in proportion of Q^4,I^. Glasses close to the Mg endmember also showed slightly higher proportions of highly depolymerized species like Q^0^ and Q^1^. XANES spectra at the Si K-edge of Na-Mg aluminosilicate glasses exhibited peaks typical of silicates glasses, and the peaks were more pronounced in the Mg endmember. Replacing Na with Mg led to the following changes:1) the K-edge shifted to higher values, which indicated increased depolymerization; 2) FWHM of the K-edge increased, implying higher structural disorder; 3) The first and strongest peak (in the Fourier transform of EXAFS spectra) shifted to higher values. This could be because of increased depolymerization. The FWHM of this peak also increased, which may be attributed to increased structural disorder.

All the glasses were subjected to alkaline dissolution to estimate their reactivities. Reactivity is a necessary, although not sufficient, requirement for a suitable precursor for AAMs. As Mg replaced Na in Na-Mg aluminosilicate glasses, the solubility of Si increased, reached a maximal value, and then decreased. Al also showed the same trend, except that it decreased at a faster pace when compared to Si. This is because of the precipitation of Al and Mg as hydrotalcite. According to XRD and STEM analysis, Si did not visibly contribute to the precipitate formation. Hence, Si solubility can used to represent the alkaline reactivity of the glasses under the far-from-equilibrium test conditions applied here. The trend in reactivity could not be explained by the conventional parameters used for estimating glass reactivity: NBO/T (which predicts similar reactivity for all glasses) and optical basicity (which predicts a direct decrease in reactivity with increasing Mg replacement). The reactivity of the studied glasses mostly depended on two factors: depolymerization and optical basicity. The effect of depolymerization dominated at lower Mg content, which led to an increase in reactivity, while the effect of optical basicity dominated in Mg-rich glasses, leading to a reduction in reactivity. Hence, while designing reactive glasses for alkali activation, glass structure should be given due consideration apart from the conventional factors such as NBO/T and optical basicity.

## Supporting information

S1 Table Peak position and FWHM used for deconvolution of Raman spectra. (DOCX)

S2 Table BET surface areas of samples. (DOCX)

S1 File Calculation of alkaline solubility of glasses. (DOCX)
