# Supplementary material for: Towards designing reactive glasses for alkali activation: Understanding the origins of alkaline reactivity of Na-Mg aluminosilicate glasses
Source: PLoS One. 2020 Dec 30;15(12):e0244621. doi: 10.1371/journal.pone.0244621 (PMC7773238; doi:10.1371/journal.pone.0244621)
Supplement: S1 Table — (DOCX) [file pone.0244621.s001.docx]

**S1 Table 1. Peak position and FWHM used for deconvolution of Raman spectra**

|  | **Q^0^** | **Q^1^** | **Q^2^** | **Q^m^** | **Q^3^** | **Q^4,II^** | **Q^4,I^** | **Carbonate** |
| --- | --- | --- | --- | --- | --- | --- | --- | --- |
| **Peak position (cm^-1^)** | 860(±3) | 900(±5) | 950(±4) | 1000(±3) | 1050(±4) | 1100(±3) | 1130(±1) | 1085 (±1) |
| **FWHM (cm^-1^)** | 45 (±3) | 65 (±4) | 65 (±2) | 73 (±2) | 65 (±2) | 53 (±2) | 47 (±3) | 25 (±1) |
