# Supplementary material for: Towards designing reactive glasses for alkali activation: Understanding the origins of alkaline reactivity of Na-Mg aluminosilicate glasses
Source: PLoS One. 2020 Dec 30;15(12):e0244621. doi: 10.1371/journal.pone.0244621 (PMC7773238; doi:10.1371/journal.pone.0244621)
Supplement: S2 Table — (DOCX) [file pone.0244621.s002.docx]

| **Sample code** | **Surface area (m^2^/g)** |
| --- | --- |
| G0.00 | 1.049 |
| G0.11 | 1.055 |
| G0.22 | 1.067 |
| G0.33 | 1.098 |
| G0.44 | 1.124 |
| G0.55 | 1.152 |
| G0.66 | 1.079 |
| G0.77 | 1.088 |
| G0.88 | 1.084 |
| G1.00 | 1.113 |

**S2 Table 1. BET surface areas of samples**
