# Supplementary material for: Towards designing reactive glasses for alkali activation: Understanding the origins of alkaline reactivity of Na-Mg aluminosilicate glasses
Source: PLoS One. 2020 Dec 30;15(12):e0244621. doi: 10.1371/journal.pone.0244621 (PMC7773238; doi:10.1371/journal.pone.0244621)
Supplement: S1 File — (DOCX) [file pone.0244621.s003.docx]

**S3 Calculation of alkaline solubility of glasses**

Normalized (w.r.t surface area of G0.00) solubility of glass “i”, $S_{i}=\frac{M_{li}}{M_{gi}}*\frac{A_{0.00}}{{Ai}}$

Where, $M_{li}=moles of silicon released into solution according to ICP analysis$

$$M_{gi}=moles of silicon in the glass beore alkali solubility test$$

$A_{i}=surface area of glass$ “i”

$$A_{0.00}=surface area of glass G0.00$$
